# Combinatorial high-throughput experimental and bioinformatic approach identifies molecular pathways linked with the sensitivity to anticancer target drugs

**DOI:** 10.18632/oncotarget.4507

**Published:** 2015-07-30

**Authors:** Larisa Venkova, Alexander Aliper, Maria Suntsova, Roman Kholodenko, Denis Shepelin, Nicolas Borisov, Galina Malakhova, Raif Vasilov, Sergey Roumiantsev, Alex Zhavoronkov, Anton Buzdin

**Affiliations:** ^1^ Drug Research and Design Department, Pathway Pharmaceuticals, Wan Chai, Hong Kong, Hong Kong SAR; ^2^ Department of Personalized Medicine, First Oncology Research and Advisory Center, Moscow, Russia; ^3^ Laboratory of Bioinformatics, D. Rogachyov Federal Research Center of Pediatric Hematology, Oncology and Immunology, Moscow, Russia; ^4^ Group for Genomic Regulation of Cell Signaling Systems, Shemyakin-Ovchinnikov Institute of Bioorganic Chemistry, Moscow, Russia; ^5^ National Research Centre “Kurchatov Institute”, Centre for Convergence of Nano-, Bio-, Information and Cognitive Sciences and Technologies, Moscow, Russia; ^6^ Pirogov Russian National Research Medical University, Department of Oncology, Hematology and Radiotherapy, Moscow, Russia; ^7^ Moscow Institute of Physics and Technology, Department of Translational and Regenerative Medicine, Dolgoprudny, Moscow Region, Russia; ^8^ Insilico Medicine, Inc, ETC, Johns Hopkins University, Baltimore, MD, USA

**Keywords:** anticancer target drugs, cancer, signaling pathway, metabolic pathway, gene expression

## Abstract

Effective choice of anticancer drugs is important problem of modern medicine. We developed a method termed OncoFinder for the analysis of new type of biomarkers reflecting activation of intracellular signaling and metabolic molecular pathways. These biomarkers may be linked with the sensitivity to anticancer drugs. In this study, we compared the experimental data obtained in our laboratory and in the Genomics of Drug Sensitivity in Cancer (GDS) project for testing response to anticancer drugs and transcriptomes of various human cell lines. The microarray-based profiling of transcriptomes was performed for the cell lines before the addition of drugs to the medium, and experimental growth inhibition curves were built for each drug, featuring characteristic IC_50_ values. We assayed here four target drugs - Pazopanib, Sorafenib, Sunitinib and Temsirolimus, and 238 different cell lines, of which 11 were profiled in our laboratory and 227 - in GDS project. Using the OncoFinder-processed transcriptomic data on ∼600 molecular pathways, we identified pathways showing significant correlation between *pathway activation strength* (PAS) and IC_50_ values for these drugs. Correlations reflect relationships between response to drug and pathway activation features. We intersected the results and found molecular pathways significantly correlated in both our assay and GDS project. For most of these pathways, we generated molecular models of their interaction with known molecular target(s) of the respective drugs. For the first time, our study uncovered mechanisms underlying cancer cell response to drugs at the high-throughput molecular interactomic level.

## INTRODUCTION

Despite the current progress in the development of innovative anticancer therapeutics, the patient's response to treatment remains largely individual, thus demanding identification of novel biomarkers predicting effectiveness of therapy for a patient. These markers may deal with specific genetic, epigenetic and gene expression features of cancer tissues [1]. Their identification was dramatically facilitated with the recent advancement of high-throughput molecular biology methods like enhanced proteomic technologies, microarray profiling of nucleic acids and next generation sequencing [2, 3]. Currently, a number of projects have been initiated to estimate the efficacy of therapeutic compounds on various cancer cell lines and link it to candidate genetic biomarkers. The first step in this field was made by National Cancer Institute (NCI) 60 human tumor cell line anticancer drug discovery project (NCI-60), profiling approximately 20000 different compounds [4, 5]. More recently, other similar projects have been developed, including Cancer Cell Line Encyclopedia project (CCLE) [4], and the collaborative Wellcome Trust Sanger-Massachusetts General Hospital Genomics of Drug Sensitivity in Cancer (GDS) project [6]. However, compared to NCI-60 these projects have lower number of profiled drugs: 24 for CCLE and 140 for GDS. Greater number of cell lines enables more in-depth analysis of drug-induced responses and identification of regulatory signatures of rare cancer subtypes. Hence, for our analysis we took GDS dataset, as it has more cell lines than NCI-60 and profiles significantly more drugs than CCLE, featuring many of the routinely used target anticancer drugs. Cellular viability was measured and inhibition curves were built for the drugs, with the characteristic half maximal inhibitory concentration (IC_50_) value measured for each cell line and each component. Overall, IC_50_ inversely correlates with the activity of a drug to inhibit cellular viability [6]. Comparison of pre-treatment gene expression patterns with the activities of certain components may be a useful tool for the identification of novel biomarkers predicting response to a therapeutic, at least at the level of cell.

However, when considering cancer markers, then general physiological processes like uncontrolled cell division, lack of feedback signaling with the enclosing normal tissues and metabolic abnormalities [7], appear to be more powerful diagnostic tools rather than expression of certain individual genes. This phenomenon may be explained by the observation that most of individual genes involved in cancerogenesis act not separately, but rather as parts of larger molecular ensembles, like molecular signaling and metabolic pathways, responsible for certain elementary molecular events [8]. Aberrations in very different individual pathway members may have similar effects on the final output of a pathway. This means that intra-pathway variation may be high, whereas overall pathway activation signature may be stable. Our recent works fully support this theoretical consideration [9]. We created a bioinformatical method termed OncoFinder for the analysis of activation of intracellular molecular pathways basing on the large-scale gene expression data [10]. The output measure is a Pathway Activation Strength (PAS), which positively reflects the degree of a pathway activation. PAS value makes it possible to quantitatively estimate the extent of each pathway activation in a given sample relative to the control sample or a set of control samples [10, 11]. OncoFinder is, to our knowledge, a unique PAS calculating method, that provides output data with significantly reduced noise introduced by the experimental gene expression platforms [12]. We showed that for most cancer types, PAS values are significantly more stable biomarkers in comparison to expression of individual genes [9]. Since the method publication in 2014, OncoFinder was applied by us and others for molecular pathway analysis in different objects including leukemia and various solid cancers [9, 13–15], Hutchinson Gilford Disease [16] and Age-Related Macular Degeneration Disease [17].

In this study, we applied PAS values to identification of enhanced biomarkers of cell response to treatment with drugs. We took four target anticancer drugs currently routinely used for renal cancer therapy: Pazopanib, Sorafenib, Sunitinib and Temsirolimus. No specific indications exists so far for making a choice which drug will be of a greater benefit for an individual patient. Here, we aimed to identify molecular pathways that correlate with the cellular response to those drugs. To this end, we took gene expression information from GDS project and compared them with the cell growth inhibition data obtained for the above four drugs. We processed gene expression data through OncoFinder to profile activation of 272 signaling and 321 metabolic molecular pathways and correlated the resulting PAS signatures with the IC_50_ values for the respective drug-cell line combinations. To validate the results, we performed similar assay in our laboratory on the experimental panel including 11 human cancer cell lines, for which we profiled transcriptomes on Illumina HT12 v4 bead arrays and established IC_50_ values for the same drugs. We intersected the results obtained with the GDS panel and in our experiments, and found a fraction of molecular pathways significantly correlated in both assays. For most of these pathways, we created molecular models of their implication with known molecular target(s) of the respective drugs. For the first time, our study uncovered mechanisms underlying cancer cell response to drugs at the high-throughput level of molecular interactions. The list of molecular pathways associated with drug response may be helpful for building prognostic tools predicting treatment option efficiencies for an individual patient in the future.

## RESULTS AND DISCUSSION

**Figure 1:**
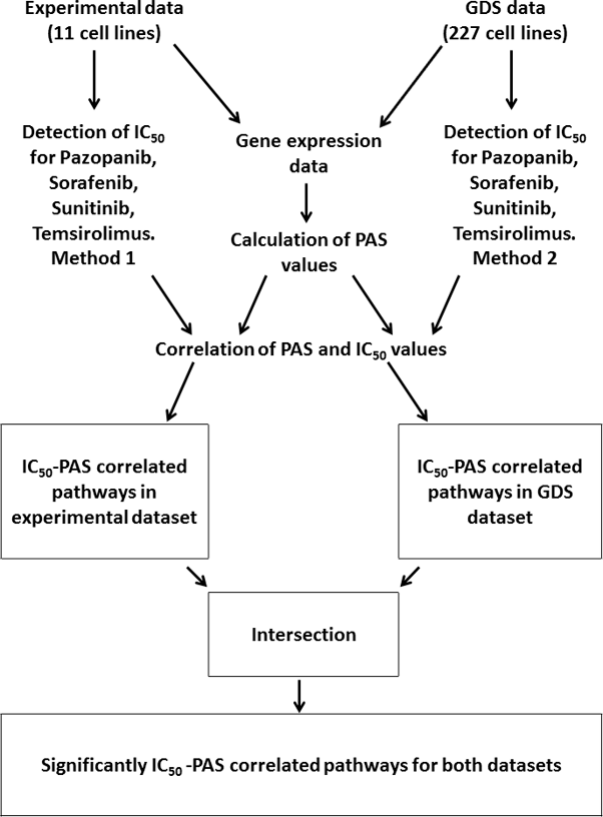
Outline of the procedures used to identify drug sensitivity-linked pathways

In this study, we for the first time compared molecular pathway activation features linked with the sensitivity of human cells to four target anticancer drugs routinely used for treatment of renal carcinoma and other cancers: Pazopanib, Sunitinib, Sorafenib and Temsirolimus. To this end, we compared pathway activation strength (PAS) signatures for experimental group of samples including eleven human cell lines grown and profiled in our laboratory, and for a database linked with “Genomics of Drug Sensitivity in Cancer” [6] project published on GDS website (http://www.cancerrxgene.org/) and including transcriptomes of 227 different human cell lines. In both projects, the half maximal inhibitory concentration (IC_50_) was measured for the above four anticancer drugs, which is a measure of the effectiveness of these drugs in inhibiting cell growth, proliferation and viability. The IC_50_ features were further compared with the PAS signatures of both experimental and GDS cell lines, and lists of molecular pathways showing significant (*p* < 0.05) correlation between PAS profiles and IC_50_ were generated. We next overlapped these lists of characteristic experimental and GDS datasets, and identified a set of molecular pathways linked with sensitivity to drugs and common to both datasets. These pathways included both intracellular signaling and metabolic pathways, and in general had multiple direct and indirect connections with the molecular targets of the respective drugs, thus explaining their association with the drug efficiency. Outline of the experimental and bioinformatic procedures utilized in this study is shown on Figure 1.

### Experimental profiling of cell transcriptomes and drug response peculiarities

In this study, we used eleven human established cell lines to profile gene expression and responses to anticancer drugs. The NT2/D1, Tera-1, NGP, HepG2, BT474, Skov-3, T3M4, HeLa, A549, Jurkat and MCF-7 cells were grown to isolate RNA and to examine their viability in the presence of anticancer target drugs Pazopanib, Sunitinib, Sorafenib and Temsirolimus. Cellular viabilities were measured using MTT test at eight different concentrations of each drug in the medium, and dose response curves were generated for each couple drug-cell line (Supplementary Dataset 1). Basing on these data, IC_50_ values were deduced for each combination. We found that for the same components, IC_50_ values differed greatly among the cell lines, showing up to 12 fold difference (Supplementary Dataset 2).

In parallel, aliquots of the respective eleven cell lines without addition of chemicals were subject to further gene expression assay. RNA was isolated, amplified and hybridized onto the bead arrays using the Illumina HT-12v4 Expression Chip (Illumina, USA). This gene expression platform contains >25,000 annotated genes and >48,000 probes derived from the National Center for Biotechnology Information RefSeq (build 36.2, release 22) and the UniGene (build 199) databases. The primary gene expression data are available at GEO repository with the accession number GSE65314. To functionally annotate primary gene expression data, we applied our original algorithm termed OncoFinder [10]. It enables calculation of the Pathway Activation Strength (PAS), *a* value which serves as a qualitative measure of pathway activation. Greater PAS value corresponds to stronger activation of a pathway, and vice versa. PAS were shown to serve as better markers of cancer progression compared to individual genes [9] and were shown to diminish discrepancies in transcriptomic data introduced by the errors of different experimental platforms, thus increasing accuracy of analyses [12]. For this algorithm, at the initial step, the transcriptome under investigation should be compared with the control set of transcriptomes to identify differentially regulated genes [10]. Overall results of such analysis depend significantly on what sample or group of samples is taken as the control. To ensure the suboptimal control will not bias the results, we applied multiple simultaneous controls for calculating PAS scores in our experiments, and took separately eleven control gene expression datasets corresponding to different normal human tissues profiled on the same platform as the experimental sampling (Illumina HT-12 arrays), 4–33 samples per dataset (Supplementary Dataset 3). These control transcriptomes were extracted from the Gene Expression Omnibus (GEO) database (http://www.ncbi.nlm.nih.gov/geo/). The PAS scores were calculated independently for all the control datasets taken one by one. The results for 272 signaling and 321 metabolic pathways were obtained for each sample, being normalized separately on each of the eleven control datasets (listed in Supplementary Dataset 4 for the experimental data).

### Analysis of cell transcriptomes and drug response information from the GDS project database

We analyzed GDS project gene expression data deposited at ArrayExpress database available at http://www.ebi.ac.uk/arrayexpress/experiments/E-MTAB-783/. This database accumulates data on gene expression in 707 human cell lines along with the corresponding IC_50_ values measured for 140 chemical components, including Pazopanib, Sunitinib, Sorafenib and Temsirolimus. For further analysis, we used the enclosing data corresponding to 227 cell lines, for which the information for these four chemicals was present. IC_50_ data were extracted and catalogued (Supplementary Datasets 5–6). In our experiments, we used MTT test to assess cell viability, whereas GDS consortium utilized alternative approach for IC_50_ profiling. Following incubation with the chemical components, cells were fixed in formaldehyde for 30 minutes and then stained with 1 μM of the fluorescent nucleic acid stain Syto60 (Invitrogen) for 1 hour. For suspension cell lines, cells were treated with compound immediately following plating, returned to the incubator for a 72 hour time point, then stained with 55 μg/ml Resazurin (Sigma) prepared in Glutathione-free media for 4 hours. Quantitation of fluorescent signal intensity was performed using a fluorescent plate reader at excitation and emission wavelengths of 630/695 nM for Syto60, and 535/595 nM for Resazurin (http://www.cancerrxgene.org/help/#t_screening). Gene expression was measured using the HT-HGU133A Affymetrix Whole Genome Array platform, raw data available online at http://www.ebi.ac.uk/arrayexpress/experiments/E-MTAB-783/protocols/. We next calculated PAS values for these transcriptomes, for the same set of signaling and metabolic pathways as for the experimental profiling. For the normalization of transcriptomes prior processing through the OncoFinder algorithm, we used three independent gene expression datasets taken from GEO database that were obtained using the same experimental platform, corresponding to three normal human tissues. Complete pathway activation data are given in Supplementary Dataset 7.

### Links between PAS signatures and drug sensitivity for the experimental data and GDS results

**Figure 2:**
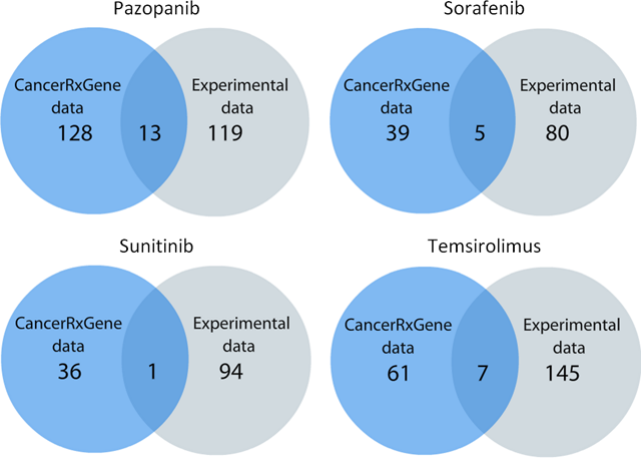
Schematic representation of the statistics on molecular pathways correlated with the response to drug treatment

To find out dependences between PAS and IC_50_ signatures, we calculated correlation coefficient values, separately for the experimental and the GDS datasets, for all the normalization methods used (Supplementary Datasets 8 and 9, respectively). The correlations were calculated according to Pearson's product moment correlation coefficient. The statistical threshold (*p* < 0.05) was used to filter significant vs non-significant correlations. We identified a number of pathways showing significant positive or negative correlation between PAS and IC_50_ values for the above four anticancer drugs (Supplementary Datasets 10 and 11 for either experimental or GDS data, respectively). A positive correlation between PAS and IC_50_ values means that the greater is the pathway activation score, the bigger is the half-inhibitory drug concentration, and the lower is the drug efficiency. Negative correlation, in contrast, means increase of the drug efficiency with the increase of PAS value. We next compared significantly correlated pathways from both datasets and found 13, 1, 5 and 7 overlapping molecular pathways for Pazopanib, Sunitinib, Sorafenib and Temsirolimus, respectively (Figure 2, Table 1).

**Table 1:** Molecular pathways correlating with drug response, overlapping for the experimental and GDS datasets

Drug	Molecular pathways	Number of normalization datasets		Sign of correlation
		GDS (out of 3)	Experimental (out of 11)	
Sorafenib	3-phosphoinositide_biosynthesis	1	5	+
Sorafenib	AKT_vPathway_Apoptosis_Inhibition	2	1	+
Sorafenib	AKT_Pathway_Elevation_of_Glucose_Import	2	1	+
Sorafenib	Androgen_receptor_Pathway_Apoptosis	2	6	+
Sorafenib	cAMP_Pathway_Metabolic_Energy	1	6	+
Sunitinib	AKT_Pathway_Protein_Synthesis	1	1	−
Pazopanib	Androgen_receptor_Pathway_Gonadotropin_Regulation	1	2	−
Pazopanib	Androgen_receptor_Pathway_Histone_Modification	1	2	−
Pazopanib	Androgen_receptor_Pathway_Prostate_Differentiation_&_Development	1	2	−
Pazopanib	Androgen_receptor_Pathway_Sexual_Differentiation_&_Sexual_Maturation_at_Puberty	1	2	−
Pazopanib	ATM_Pathway	3	3	+
Pazopanib	zymosterol_biosynthesis	2	1	+
Pazopanib	SMAD_m_Pathway_Degradation	1	9	+
Pazopanib	CD40_Pathway_Cell_Survival	3	1	+
Pazopanib	chondroitin_sulfate_biosynthesis_late_stages	1	1	−
Pazopanib	Circadian_Pathway	2	1	+
Pazopanib	dermatan_sulfate_biosynthesis_late_stages	3	1	−
Pazopanib	SMAD_m_Pathway_Degradation	1	9	+
Pazopanib	spermidine_biosynthesis	1	3	−
Pazopanib	triacylglycerol_biosynthesis	1	1	+
Temsirolimus	purine_deoxyribonucleosides_degradation	3	1	−
Temsirolimus	RAS_Pathway	1	1	−
Temsirolimus	GSK3_Pathway_Gene_Expression	2	11	−
Temsirolimus	phytol_degradation	1	2	+
Temsirolimus	tryptophan_degradation_ mammalian_via_tryptamine	2	7	+
Temsirolimus	cAMP_Pathway_Glycogen_Synthesis	2	1	−
Temsirolimus	D-imyoi-inositol_1, 4, 5-trisphosphate_degradation	2	6	+

### Pazopanib

Pazopanib, also known as Votrient, is a tyrosine kinase inhibitor that targets proteins VEGFR-1, VEGFR-2, VEGFR-3, PDGFR-a/β and c-kit. For Pazopanib, there were identified 4 and 2 positively correlated, and 4 and 3 negatively correlated signaling and metabolic pathways, respectively (Table 1). All negatively correlated signaling pathways represented brunches of Androgen receptor signalization, four positives were brunches of CD40, ATM, Circadian clock and SMAD pathways. Negative correlation of Androgen signaling means that its increase coincides with greater sensitivity to Pazopanib. This observation is in line with previously reported fail of clinical trials of Pazopanib in castrate-sensitive (androgen signaling-negative) prostate cancer patients [18]. For positively-correlated pathways, we found a recent literature report that Pazopanib most likely suppresses cell cycle progression in cancer cells by preventing inactivation of ATM checkpoint signaling [19]. Thus, enhanced activity of Pazopanib may be linked with dynamic trans-activation of ATM, which is originally suppressed in a cancer cell, in good agreement with the positive correlation discovered here. No previous reports were found for links between the activities of Pazopanib and CD40, Circadian clock and SMAD signaling, and for all metabolic pathways.

### Sunitinib

Sunitinib (Sutent), is a tyrosine kinase inhibitor that targets proteins FLT1, FLT3, FLT4, c-kit, PDGFR-a/β, and KDR. For this drug, we found a unique negatively correlated pathway representing a brunch of AKT signaling responsible for protein synthesis regulation (Table 1). Numerous studies indicate that Sunitinib acts by suppressing AKT signaling in many ways (e.g., [20–23]). AKT signaling-positive cancer cells, therefore, may be good targets for treatment with Sunitinib, whereas the negative cells may be worse responding candidates, in good agreement with our findings. Moreover, co-suppression of protein biosynthesis pathway by inhibiting mTOR using Rapamycin, showed a significant synergistic effect with Sunitinib against cell proliferation [24].

### Sorafenib

Sorafenib (Nexavar), is a kinase inhibitor drug that targets proteins PDGFR-a/β, FLT3, RET, BRAF, KDR, FLT4, RAF1, FLT1, FGFR, and c-kit. For Sorafenib, we identified only five positively correlated pathways: 4 signaling and 1 metabolic pathways, respectively (Table 1). Two signaling pathways represented brunches of AKT, one—of cAMP, and the last one—of Androgen receptor signalization. The only correlated metabolic pathway deals with the 3′-phosphoinositide biosynthesis.

AKT pathway extensions responsible for the inhibition of apoptosis and for the elevation of glucose uptake, appeared to be positively correlated with Sorafenib activity. This means that their upregulation interferes with the efficiency of Sorafenib treatment. The interference of AKT-induced glucose uptake with the activity of Sorafenib was recently mentioned in the literature [25]. In turn, activation of AKT and consequent escape of apoptosis is the mechanism of resistance of hepatocellular carcinoma cells to Sorafenib [26]. Similarly, compensatory activation of AKT was identified as one of major reasons hampering Sorafenib activity also in urothelial cells [27]. In both cases, the authors noted that AKT signaling worked through the compensatory activation of the phosphatidylinositol-3-kinase (PI3K) pathway [26, 27]. In light of these findings, it is particularly interesting that the only metabolic pathway that was positively correlated with Sorafenib IC_50_ in our study appeared to be a pathway responsible for the 3′-phosphoinositide biosynthesis, which is tightly associated with the above PI3K signaling (Table 1). Androgen receptor-controlled suppression of apoptosis, along with the AKT pathway, are known as the major targets of Sorafenib in prostate cancer cells [28]. Finally, the mutually interfering effects of cAMP signaling promoting cell growth, enhanced metabolism and proliferation, and of Sorafenib, were documented previously for renal epithelial cells [29].

### Temsirolimus

Temsirolimus is a small molecule that targets FRAP1 protein, also known as mTOR. For Temsirolimus, we identified 3 positively correlated metabolic pathways, and 3 and 1 negatively correlated signaling and metabolic pathways, respectively (Table 1). The activation of negatively correlated pathways largely coincides with the enhanced activity of Temsirolimus, and the contrary is true for the positively correlated pathways. The negatively correlated signaling pathways include RAS pathway, cAMP pathway-regulated Glycogen synthesis, and a terminal brunch of GSK3 pathway regulating gene expression. The only negatively correlated metabolic pathway was the pathway of Purine deoxyribonucleosides degradation, and the positively correlated metabolic pathways were pathways of D-myo-inositol 1,4,5-trisphosphate degradation, Phytol degradation and Tryptophan degradation via tryptamine. For those pathways, we found no literature reports linking them with the activity of Temsirolimus.

## CONCLUSION

**Figure 3:**
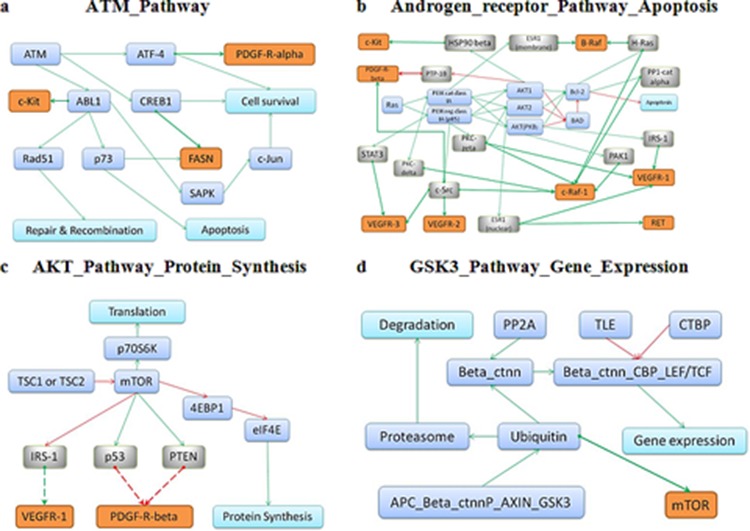
Schematic representation of the respective drug targets in the overall architecture of molecular interactions for the top pathways correlating with response to Pazopanib **A.** Sorafenib **B.** Sunitinib **C.** and Temsirolimus **D.** Protein targets of the respective drugs are shown in orange, intermediate molecules between pathway members and drug targets (in grey) and pathway members (in blue).

Importantly, the molecular pathways that overlapped between our cell culture assay and GDS data, were identified to be significantly linked with the response to drugs in two independent experimental cell viability tests performed in two different laboratories. For those pathways, we attempted to find out functional relationships between pathway members and known molecular targets of the above-mentioned respective drugs. To this end, we used Metacore knowledgebase (Thompson Reuters, USA) and identified 20 direct and 145 indirect molecular interactions that link the pathways with related drug targets, for all tested drugs (Supplementary Dataset 12). In most cases, these interactions explain the involvement of pathways identified in drug response. The outline depicting interactions of drugs with their targets for top molecular pathways is shown on Figure 3 for Pazopanib (Figure 3a), Sorafenib (Figure 3b), Sunitinib (Figure 3c) and Temsirolimus (Figure 3d). In our study, we identified several previously unknown connections between intracellular molecular signaling and drug efficiency. We note that links between cancer and metabolic pathways are still poorly understood relatively to those for the intracellular signalization pathways. The data obtained here may be valuable for design of novel therapeutic strategies supplementing treatment with the above anticancer drugs by the additional components targeting relevant molecular pathways. In the future, similar approach may be applied also for assessing the effects linked with resistance to radiation therapy [30]. Provided that activation of molecular pathways may serve as a superior biomarker relatively to expression of enclosing individual gene products, we conclude, that additional coordinated high-throughput studies are needed to explore the currently underinvestigated galaxy of pathway-drug interactions.

## MATERIALS AND METHODS

### Cell culture

In this study, we used eleven human cell lines to profile gene expression and responses to anticancer drugs. The NT2/D1, Tera-1, NGP, HepG2, BT474, Skov-3, T3M4, HeLa, A549, MCF-7 cells were cultured on Dulbecco's modified Eagle's medium (DMEM) (PanEco, Russia) supplemented with 10% fetal calf serum (HyClone, USA), 100 mcg/ml penicillin (Sigma, USA), 100 U/ml streptomycin (Sigma, USA) and 2mM L-glutamine (Sigma, USA) at 37°C and 5% CO_2_. Jurkat cells were maintained in RPMI-1640 medium (PanEco, Russia) with the same supplements. The cells were grown in 25 cm^2^ or 75 cm^2^ flask (Greiner, Germany) and passaged for every 72 hours.

### Cell viability assay

We evaluated cell viability by using MTT (3-[4,5-dimethylthiazol-2-yl]-2,5-diphenyltetrazolium bromide) test [31]. Adherent cells were dissociated from plastic vessel using trypsin-EDTA solution (PanEco, Russia), then cells were washed twice with DMEM/RPMI-1640. Aliquots of cells were counted in hemocytometer. Cells were inoculated in 96-well plates (Greiner, Germany), ~2.000–10.000 cells per well, depending on the cell line used. The plates were pre-incubated for 18 hr before the addition of testing components. The following drugs were tested (purchased at Selleckchem, USA): Pazopanib, Sunitinib, Sorafenib and Temsirolimus. For every cell line, the drugs were tested in the following concentrations: 0, 0.8, 1.56, 3.1, 6.25, 12.5, 25 and 50 μM. The chemicals were added to the culture medium in DMSO solution. All the experiments were made in quadruplicate. After addition of the testing components, the plates were incubated for 72 hr and then centrifuged at 300 g for 10 min using plate centrifuge (Biosan, Latvia), followed by the removal of supernatant. 30 μl of 0.5 mg/ml solution of MTT (Sigma, USA) was added to each well, and the plates were incubated for 2–4 hr, depending on the cell line used, then 100 μl of DMSO was added to each well and mixed by pipetting until all blue formazan crystals were dissolved. The optical densities (OD) of each well were measured using a plate reader Multiscan FC (ThermoScientific, USA) at 540 nm wavelength. Cell viability was calculated using the formulae: (OD treated well – OD blank)/(mean OD control well – OD blank) × 100%, where OD blank means OD in control wells containing no cells. IC_50_ values were deduced from Dose-response curves using SigmaPlot software (Systat Software Inc., USA). Dose-response curves are given in Supplementary Dataset 1. The experimentally measured IC_50_ values are shown on Supplementary Dataset 2.

### Experimental gene expression analysis

Approx. 0.5 million cell aliquots of the respective eleven cell lines without addition of chemicals were subject to further gene expression assay. RNA was isolated using TRIzol Reagent (Life Technologies, USA) following the manufacturer's protocol. Purified RNA was dissolved in RNase-free water and stored at −80°C. RNA was then reverse-transcribed to cDNA and cRNA using the Ambion TotalPrep cRNA Amplification Kit (Invitrogen, USA). The cRNA concentration was quantified and adjusted to 150 ng/ml using an ND-1000 Spectrophotometer (NanoDrop Technologies, USA). A total 750 ng of each RNA library was hybridized onto the bead arrays. Gene expression experiments were performed by Genoanalytica (Moscow, Russia) using the Illumina HumanHT-12v4 Expression BeadChip (Illumina, Inc.). This gene expression platform contains more than 25,000 annotated genes and more than 48,000 probes derived from the National Center for Biotechnology Information RefSeq (build 36.2, release 22) and the UniGene (build 199) databases. The primary gene expression data are available through GEO repository with the accession number GSE65314.

### Database gene expression data

We analyzed gene expression datasets deposited in ArrayExpress database available at http://www.ebi.ac.uk/arrayexpress/experiments/E-MTAB-783/. This database accumulates data on gene expression in 707 human cell lines along with the corresponding IC_50_ values, deposited in The Genomics of Drug Sensitivity in Cancer (GDS) database, available at http://www.cancerrxgene.org/downloads/, measured for 140 chemical components, including Pazopanib, Sunitinib, Sorafenib and Temsirolimus. In the GDS project database, we found matching transcriptomic and IC_50_ information, corresponding to the above four drugs, for 227 cell lines. In GDS project, gene expression was measured using HT-HGU133A Affymetrix Whole Genome Array platform.

### Pathway activation analysis

For the functional annotation of the primary gene expression data, we applied our original algorithm termed OncoFinder [6, 8, 9]. It enables calculation of the Pathway Activation Strength (PAS), *a* value which serves as a qualitative measure of pathway activation. Briefly, the enclosing algorithm utilizes the following formula to evaluate pathway activation:

$${\text{PAS}}_{p}=\sum_{n}{\text{ARR}}_{\text{np}}\cdot {\text{BTIF}}_{n}\cdot \text{lg}({\text{CNR}}_{n})$$

Here the *case-to-normal ratio*, *CNRn*, is the ratio of expression levels for a gene *n* in the sample under investigation to the same average value for the control group of samples. The Boolean flag of *BTIF* (*beyond tolerance interval flag*) equals to zero when the *CNR* value has passed simultaneously the two criteria that demark the significantly perturbed expression level from essentially normal: first, the expression level for the sample lies within the tolerance interval, where *p* > 0.05, and second, the value of *CNR* differs from 1 considerably, *CNR* 0.66 or *CNR* 1.5. The discrete value of *ARR* (*activator / repressor role*) reflects the functional role of a gene product n in the pathway [8, 9]. For quantile normalization of gene expression in our experimental data (eleven cell lines), we used separately another eleven gene expression datasets corresponding to sets of different normal human tissues profiled on Illumina HT-12v3-4 platforms, 4–33 samples per each dataset (Supplementary Dataset 3). For quantile normalization of the GDS data, we used three gene expression datasets obtained using the platform Affymetrix HT-HGU133A whole genome array, corresponding to three normal human tissues, 2–10 samples per each dataset (Supplementary Dataset 7). The results for 272 signaling and 321 metabolic pathways were obtained for each sample (details shown on Supplementary Dataset 4 for our original experimental data and on Supplementary Dataset 7 for the GDS data).

### Statistical tests

The correlations between PAS and IC_50_ values were calculated according to Pearson's product moment correlation coefficient. The statistical threshold (*p* < 0.05) was used to filter significant vs non-significant correlations. We used test for association between paired samples and function *cor.test* (https://stat.ethz.ch/R-manual/R-patched/library/stats/html/cor.test.html) in R (http://www.r-project.org/) to return correlation coefficients. The full data on correlations between pathway activation and IC_50_ values, for both experimental and GDS datasets, are shown, respectively, on Supplementary Datasets 8 and 9.

### Analysis of the interactome databases

In this study, we did literature search of the NCBI PubMed database in order to manually examine pathways connected with drug response. To identify additional targets for pathway-linked regulation, we used a manually curated commercial database GeneGo (MetaCore package, Thomson Reuters, USA), and the MetaCore pathway analysis tool to visualize molecular interactions between the proteins. The manually curated functional molecular links between the top pathways and IC_50_ values are shown on Supplementary Dataset 12.

## SUPPLEMENTARY DATASETS




















