# Supplementary material for: Combinatorial high-throughput experimental and bioinformatic approach identifies molecular pathways linked with the sensitivity to anticancer target drugs
Source: Oncotarget. 2015 Jul 30;6(29):27227–38. doi: 10.18632/oncotarget.4507 (PMC4694985; doi:10.18632/oncotarget.4507)
Supplement: Supplementary file 1 [file oncotarget-06-27227-s001.pdf]

SUPPLEMENTARY DATASETS

Supplementary Dataset S1: Dose response curves (MTT-test) for NT2/D1, Tera-1, NGP, HepG2, BT474, Skov-3, T3M4, HeLa, A549, Jurkat, MCF-7 cells and drugs Pazopanib, Sunitinib, Sorafenib and Temsirolimus

Supplementary Dataset S2: Experimentally measured IC_{50} values for the cell lines under investigation

Cell line name	Pazopanib IC_{50}, µM	Sorafenib IC_{50}, µM	Sunitinib IC_{50}, µM	Temsirolimus IC_{50}, µM
A549	28	7.8	4.1	18
BT474	50	14.4	7.8	22.8
HeLa	24	9.6	3.9	14.7
HepG2	29	8.1	4.5	23.2
Jurkat	3.9	6	2.3	4.4
MCF	50	9.7	6.4	16.3
NGP	50	15.2	7.9	15.3
NT2/D1	41.7	22.7	14	18.3
Skov-3	7.9	10.3	4.3	13.8
T3M4	29.5	9.3	4.7	11.3
Tera1	8.3	6.4	3.6	11.6

Supplementary Dataset S3: Normalization datasets used for the processing of the experimental and CancerRxGene transcriptomic data

Normalization datasets used for the processing of the experimental transcriptomic data				
**GEO dataset ID**	**Tissue type**	**Platform**	**Number of samples**	**Sample IDs**
GSE17822	Meibomian glands	Illumina Human HT-12 expression beadchip platform, V3.0	6	GSM444688, GSM444689, GSM444690, GSM444691, GSM444692, GSM444693
GSE26852	Muscle	Illumina Human HT-12 expression beadchip platform, V3.0	7	GSM661123, GSM661124, GSM661125, GSM661126, GSM661127, GSM661128, GSM661130
GSE32124	Breast	Illumina Human HT-12 expression beadchip platform, V3.0	33	GSM796359, GSM796360, GSM796361, GSM796362, GSM796363, GSM796364, GSM796365, GSM796366, GSM796367, GSM796368, GSM796369, GSM796370, GSM796371, GSM796372, GSM796373, GSM796374, GSM796375, GSM796376, GSM796377, GSM796378, GSM796379, GSM796380, GSM796381, GSM796382, GSM796383, GSM796384, GSM796385, GSM796386, GSM796387, GSM796388, GSM796389, GSM796390, GSM796391
GSE34074	Dermal fibroblasts	Illumina Human HT-12 expression beadchip platform, V3.0	6	GSM841415, GSM841419, GSM841423, GSM841427, GSM841431, GSM841435
GSE37013	Jejunum	Illumina Human HT-12 expression beadchip platform, V3.0	7	GSM908514, GSM908515, GSM908516, GSM908517, GSM908518, GSM908519, GSM908520
GSE40645	Vastus lateralis muscles	Illumina Human HT-12 expression beadchip platform, V3.0	14	GSM998797, GSM998798, GSM998799, GSM998800, GSM998801, GSM998802, GSM998803, GSM998804, GSM998805, GSM998806, GSM998807, GSM998808, GSM998809, GSM998810
GSE42656	Cerebellum	Illumina Human HT-12 expression beadchip platform, V3.0	4	GSM1047460, GSM1047462, GSM1047463, GSM1047467
GSE52130	Oral epithelium	Illumina Human HT-12 expression beadchip platform, V4.0	7	GSM1260102, GSM1260103, GSM1260104, GSM1260105, GSM1260106, GSM1260107, GSM1260108
GSE54563	Anterior cingulate cortex	Illumina Human HT-12 expression beadchip platform, V3.0	25	GSM1318920, GSM1318921, GSM1318922, GSM1318923, GSM1318924, GSM1318925, GSM1318926, GSM1318927, GSM1318928, GSM1318929, GSM1318930, GSM1318931, GSM1318932, GSM1318933, GSM1318934, GSM1318935, GSM1318936, GSM1318937, GSM1318938, GSM1318939, GSM1318940, GSM1318941, GSM1318945, GSM1318946, GSM1318947

(*Continued*)

Normalization datasets used for the processing of the experimental transcriptomic data				
**GEO dataset ID**	**Tissue type**	**Platform**	**Number of samples**	**Sample IDs**
GSE57218	Cartilage	Illumina Human HT-12 expression beadchip platform, V3.0	6	GSM1380887, GSM1380888, GSM1380956, GSM1380957, GSM1380958, GSM1380959
GSE60709	Skin	Illumina Human HT-12 expression beadchip platform, V3.0	14	GSM1486160, GSM1486161, GSM1486162, GSM1486163, GSM1486164, GSM1486165, GSM1486166, GSM1486167, GSM1486168, GSM1486169, GSM1486170, GSM1486171, GSM1486172, GSM1486173
**Normalization datasets used for the processing of the GDS transcriptomic data**				
GSE14520	Liver	Affymetrix HT Human Genome U133A Array	10	GSM362995, GSM362996, GSM362997, GSM362998, GSM362999, GSM363000, GSM363001, GSM363002, GSM363003, GSM363004
GSE14805	Gliosis non-neoplastic control tissue	Affymetrix HT Human Genome U133A Array	4	GSM370578, GSM370579, GSM370580, GSM370581
GSE21212	Aortic smooth muscle cells	Affymetrix HT Human Genome U133A Array	2	GSM530379, GSM530381

Supplementary Dataset S4: The PAS scores of experimental data which calculated independently for all the control datasets taken one by one. The results for the 272 signaling and 321 metabolic pathways were obtained for each sample, being normalized separately on each of the 11 control datasets

Supplementary Dataset S5: Statistics of the IC_{50} data extracted from GDS database from the section "Genomics of Drug Sensitivity in Cancer" for 227 human cell lines

IC_{50}, µM	Pazopanib	Sorafenib	Sunitinib	Temsirolimus
Minimal value	0.00003	0.004	0.000002	0.00001
Average value	239.9	127.2	154.6	2.2
Maximal value	1947.6	1278.3	1951.4	22.5

Supplementary Dataset S6: IC_{50} (GDS data) for Pazopanib, Sunitinib, Sorafenib and Temsirolimus, calculated for 227 cell lines

Supplementary Dataset S7: The PAS scores of CancerRxGene data which calculated independently for all the control datasets taken one by one. The results for the 272 signaling and 321 metabolic pathways were obtained for each sample, being normalized separately on each of the 3 control datasets

Supplementary Dataset S8: Correlation coefficient and *p*-value for experimental data, calculated independently for 11 control datasets

Supplementary Dataset S9: Correlation coefficient and *p*-value for GDS data, calculated independently for 3 control datasets

Supplementary Dataset S10: Molecular pathways displaying significant correlations between PAS and IC_{50} signatures in experimental dataset obtained in this study. Linked positive and negative correlations are shown for all used normalization datasets

Supplementary Dataset S11: Molecular pathways displaying significant correlations between PAS and IC_{50} signatures in GDS dataset. Linked positive and negative correlations are shown for all normalization datasets used

Supplementary Dataset S12: Possible ways of interactions between the overlapping molecular pathway components and the respective drug targets
