# Supplementary material for: Combinatorial high-throughput experimental and bioinformatic approach identifies molecular pathways linked with the sensitivity to anticancer target drugs
Source: Oncotarget. 2015 Jul 30;6(29):27227–38. doi: 10.18632/oncotarget.4507 (PMC4694985; doi:10.18632/oncotarget.4507)
Supplement: Supplementary file 2 [file oncotarget-06-27227-s002.docx]

**Supplementary file 1. Dose response curves (MTT-test) for NT2/D1, Tera-1, NGP, HepG2, BT474, Skov-3, T3M4, HeLa, A549, Jurkat, MCF-7 cells and drugs Pazopanib, Sunitinib, Sorafenib and Temsirolimus**.

A-549

| A-549, Pazopanib | | |
| --- | --- | --- |
| date | 26-28.4.14. | 5-8.5.14. |
| IC50 | 24 | 24 |
|  |  |  |
|  |  |  |
| A-549, Sorafenib | | |
| date | 26-28.4.14. | 5-8.5.14. |
| IC50 | 8.5 | 7 |
|  |  |  |
|  |  |  |
| A-549, Sunitinib | | |
| date | 25-28.4.14. | 5-8.5.14. |
| IC50 | 5.6 | 2.5 |
|  |  |  |
|  |  |  |
| A-549, Temsirolimus | | |
| date | 26-28.4.14. | 5-8.5.14. |
| IC50 | 17 | 19 |
|  |  |  |

BT-474

| BT-474, Pazopanib | | |
| --- | --- | --- |
| date | 26-28.4.14. | 19-22.5.14 |
| IC50 | >50 | >50 |
|  |  |  |
|  |  |  |
| BT-474, Sorafenib | | |
| date | 26-28.4.14. | 19-22.5.14 |
| IC50 | 15.8 | 13 |
|  |  |  |
|  |  |  |
| BT-474, Sunitinib | | |
| date | 25-28.4.14. | 19-22.5.14 |
| IC50 | 8.5 | 7 |
|  |  |  |
|  |  |  |
| BT-474, Temsirolimus | | |
|  | 26-28.4.14. | 19-22.5.14 |
|  | 21.6 | 24 |
|  |  |  |

HeLa

| HeLa, Pazopanib | | |
| --- | --- | --- |
| date | 18-21.7.14. | 21-24.7.14. |
| IC50 | 24 | 25 |
|  |  |  |
|  | 25-28.7.14 |  |
| date | 23 |  |
| IC50 |  |  |
|  |  |  |
| HeLa, Sorafenib | | |
| date | 18-21.7.14. | 21-24.7.14. |
| IC50 | 7 | 11.5 |
|  |  |  |
| date | 25-28.7.14 |  |
| IC50 | 10.4 |  |
|  |  |  |
|  |  |  |
| HeLa, Sunitinib | | |
| date | 18-21.7.14. | 21-24.7.14. |
| IC50 | 2.7 | 6 |
|  |  |  |
| date | 25-28.7.14 |  |
| IC50 | 3.2 |  |
|  |  |  |
|  |  |  |
| HeLa, Temsirolimus | | |
| date | 18-21.7.14. | 21-24.7.14. |
| IC50 | 10 | 19.5 |
|  |  |  |
| date | 25-28.7.14 |  |
| IC50 | 15.6 |  |
|  |  |  |

HepG2

| HepG2, Pazopanib | | |
| --- | --- | --- |
| date | 26-30.6.14 | 30.6-2.7.14 |
| IC50 | 33 | 28 |
|  |  |  |
| date | 8-11.7.14 |  |
| IC50 | 26 |  |
|  |  |  |
|  |  |  |
| HepG2, Sorafenib | | |
| date | 26-30.6.14 | 30.6-2.7.14 |
| IC50 | 5.8 | 10 |
|  |  |  |
| date | 8-11.7.14 |  |
| IC50 | 8.4 |  |
|  |  |  |
|  |  |  |
| HepG2, Sunitinib | | |
| date | 26-30.6.14 | 30.6-2.7.14 |
| IC50 | 3.1 | 6.5 |
|  |  |  |
| date | 8-11.7.14 |  |
| IC50 | 4.2 |  |
|  |  |  |
|  |  |  |
| HepG2, Temsirolimus | | |
| date | 26-30.6.14 | 30.6-2.7.14 |
| IC50 | 21.5 | 23 |
|  |  |  |
| date | 8-11.7.14 |  |
| IC50 | 25 |  |
|  |  |  |

Jurkat

| Jurkat, Pazopanib | | |
| --- | --- | --- |
| date | 16-19.5.14 | 25-28.7.14 |
| IC50 | 4.8 | 2.2 |
|  |  |  |
| date | 29.7-1.8.14 |  |
| IC50 | 4.9 |  |
|  |  |  |
|  |  |  |
| Jurkat, Sorafenib | | |
| date | 16-19.5.14 | 25-28.7.14 |
| IC50 | 8.8 | 5 |
|  |  |  |
| date | 29.7-1.8.14 |  |
| IC50 | 4.2 |  |
|  |  |  |
|  |  |  |
| Jurkat, Sunitinib | | |
| date | 16-19.5.14 | 25-28.7.14 |
| IC50 | 3.1 | 2 |
|  |  |  |
| date | 29.7-1.8.14 |  |
| IC50 | 1.8 |  |
|  |  |  |
|  |  |  |
| Jurkat, Temsirolimus | | |
| date | 16-19.5.14 | 25-28.7.14 |
| IC50 | 5.1 | 3.3 |
|  |  |  |
| date | 29.7-1.8.14 |  |
| IC50 | 4.8 |  |
|  |  |  |

MCF-7

| MCF-7, Pazopanib | | |
| --- | --- | --- |
| date | 9-12.6.14 | 16-18.6.14. |
| IC50 | 50 | >50 |
|  |  |  |
| date |  |  |
| IC50 | >50 |  |
|  |  |  |
|  |  |  |
| MCF-7, Sorafenib | | |
| date | 9-12.6.14 | 16-18.6.14. |
| IC50 | 11 | 10 |
|  |  |  |
| date |  |  |
| IC50 | 8.4 |  |
|  |  |  |
|  |  |  |
| MCF-7, Sunitinib | | |
| date | 9-12.6.14 | 16-18.6.14. |
| IC50 | 6 | 7.8 |
|  |  |  |
| date | 14-17.7.14 |  |
| IC50 | 5.5 |  |
|  |  |  |
|  |  |  |
| MCF-7, Temsirolimus | | |
| date | 9-12.6.14 | 16-18.6.14. |
| IC50 | 14.6 | 16.5 |
|  |  |  |
| date |  |  |
| IC50 | 17.8 |  |
|  |  |  |

NGP

| NGP, Pazopanib | | |
| --- | --- | --- |
| date | 26-30.6.14 | 30.6-2.7.14 |
| IC50 | >50 | >50 |
|  |  |  |
| date | 7-10.7.14 |  |
| IC50 | >50 |  |
|  |  |  |
|  |  |  |
| NGP, Sorafenib | | |
| date | 26-30.6.14 | 30.6-2.7.14 |
| IC50 | 14.5 | 15 |
|  |  |  |
| date | 7-10.7.14 |  |
| IC50 | 16 |  |
|  |  |  |
|  |  |  |
| NGP, Sunitinib | | |
| date | 26-30.6.14 | 30.6-2.7.14 |
| IC50 | 8.5 | 10 |
|  |  |  |
| date | 7-10.7.14 |  |
| IC50 | 5.2 |  |
|  |  |  |
|  |  |  |
| NGP, Temsirolimus | | |
| date | 26-30.6.14 | 30.6-2.7.14 |
| IC50 | 15.5 | 18 |
|  |  |  |
| date | 7-10.7.14 |  |
| IC50 | 12.5 |  |
|  |  |  |

NT2/D1

| NT2/D1, Pazopanib | | |
| --- | --- | --- |
| date | 16-18.6.14. | 23.6-26.6.14 |
| IC50 | 25 | >50 |
|  |  |  |
| date | 11-14.7.14 |  |
| IC50 | >50 |  |
|  |  |  |
| NT2/D1, Sorafenib | | |
| date | 16-18.6.14. | 23.6-26.6.14 |
| IC50 | 23 | 20 |
|  |  |  |
| date | 11-14.7.14 |  |
| IC50 | 26 |  |
|  |  |  |
|  |  |  |
| NT2/D1, Sunitinib | | |
| date | 16-18.6.14. | 23.6-26.6.14 |
| IC50 | 15.5 | 15 |
|  |  |  |
| date | 11-14.7.14 |  |
| IC50 | 11 |  |
|  |  |  |
|  |  |  |
| NT2/D1, Temsirolimus | | |
| date | 16-18.6.14. | 23.6-26.6.14 |
| IC50 | 18.5 | 19.5 |
|  |  |  |
| date | 11-14.7.14 |  |
| IC50 |  |  |
|  |  |  |

Skov-3

| Skov-3, Pazopanib | | |
| --- | --- | --- |
| date | 21-23.7.14 | 25-28.7.14 |
| IC50 | 10 | 7.8 |
|  |  |  |
| date | 28-31.7.14 |  |
| IC50 | 6 |  |
|  |  |  |
|  |  |  |
| Skov-3, Sorafenib | | |
| date | 21-23.7.14 | 25-28.7.14 |
| IC50 | 12 | 11.3 |
|  |  |  |
| date | 28-31.7.14 |  |
| IC50 | 7.5 |  |
|  |  |  |
| Skov-3, Sunitinib | | |
| date | 21-23.7.14 | 25-28.7.14 |
| IC50 | 5.8 | 3.8 |
|  |  |  |
| date | 28-31.7.14 |  |
| IC50 | 3.2 |  |
|  |  |  |
|  |  |  |
| Skov-3, Temsirolimus | | |
| date | 21-23.7.14 | 25-28.7.14 |
| IC50 | 16.7 | 14.8 |
|  |  |  |
| date | 28-31.7.14 |  |
| IC50 | 10 |  |
|  |  |  |

T3M4

| T3M4, Pazopanib | | |
| --- | --- | --- |
| date | 5-8.5.14. | 12-15.5.14 |
| IC50 | 31 | 28 |
|  |  |  |
|  |  |  |
| T3M4, Sorafenib | | |
| date | 5-8.5.14. | 12-15.5.14 |
| IC50 | 9.4 | 9 |
|  |  |  |
|  |  |  |
| T3M4, Sunitinib | | |
| date | 5-8.5.14. | 12-15.5.14 |
| IC50 | 5.3 | 4 |
|  |  |  |
|  |  |  |
| T3M4, Temsirolimus | | |
| date | 5-8.5.14. | 12-15.5.14 |
| IC50 | 10.5 | 12 |
|  |  |  |

Tera1

| Tera1, Pazopanib | | |
| --- | --- | --- |
| date | 19-22.5.14 | 30.5.14 |
| IC50 | 12 | 10 |
|  |  |  |
| date | 22.7.14 | 29.7.14 |
| IC50 | 6.2 | 5 |
|  |  |  |
|  |  |  |
| Tera1, Sorafenib | | |
| date | 19-22.5.14 | 30.5.14 |
| IC50 | 6.5 | 3.1 |
|  |  |  |
| date | 22.7.14 | 29.7.14 |
| IC50 | 10.6 | 5.4 |
|  |  |  |
|  |  |  |
| Tera1, Sunitinib | | |
| date | 19-22.5.14 | 30.5.14 |
| IC50 | 4.4 | 3.7 |
|  |  |  |
| date | 22.7.14 | 29.7.14 |
| IC50 | 4.4 | 1.8 |
|  |  |  |
|  |  |  |
| Tera1, Temsirolimus | | |
| date | 19-22.5.14 | 30.5.14 |
| IC50 | 25 | 4.2 |
|  |  |  |
| date | 22.7.14 | 29.7.14 |
| IC50 | 10.6 | 6.7 |
|  |  |  |
